# Ethyl 2-(4-chloro­phen­yl)-1-phenyl-1*H*-benzimidazole-5-carboxyl­ate

**DOI:** 10.1107/S1600536812022192

**Published:** 2012-05-23

**Authors:** Yeong Keng Yoon, Elumalai Manogaran, Tan Soo Choon, Suhana Arshad, Ibrahim Abdul Razak

**Affiliations:** a Institute for Research in Molecular Medicine, Universiti Sains Malaysia, Minden 11800, Penang, Malaysia; b Department of Pharmaceutical Sciences, UCSI University, Kuala Lumpur, Cheras 56000, Malaysia; c School of Physics, Universiti Sains Malaysia, 11800 USM, Penang, Malaysia

## Abstract

In the title compound, C_22_H_17_ClN_2_O_2_, the essentially planar benzimidazole ring system [maximum deviation = 0.012 (2) Å] forms dihedral angles of 28.69 (6) and 63.65 (7)°, respectively, with the phenyl and chloro-substituted benzene rings. The dihedral angle between the phenyl and benzene rings is 64.23 (8)°. In the crystal, mol­ecules are linked into a zigzag chain along the *a* axis by inter­molecular C—H⋯O hydrogen bonds. C—H⋯π inter­actions are also present.

## Related literature

For applications of benzimidazoles, see: Tanious *et al.* (2004 ▶); Townsend & Revankar (1970 ▶). For related structures, see: Yoon *et al.* (2011 ▶, 2012 ▶); Kassim *et al.* (2012 ▶). For stability of the temperature controller used in the data collection, see: Cosier & Glazer (1986 ▶).
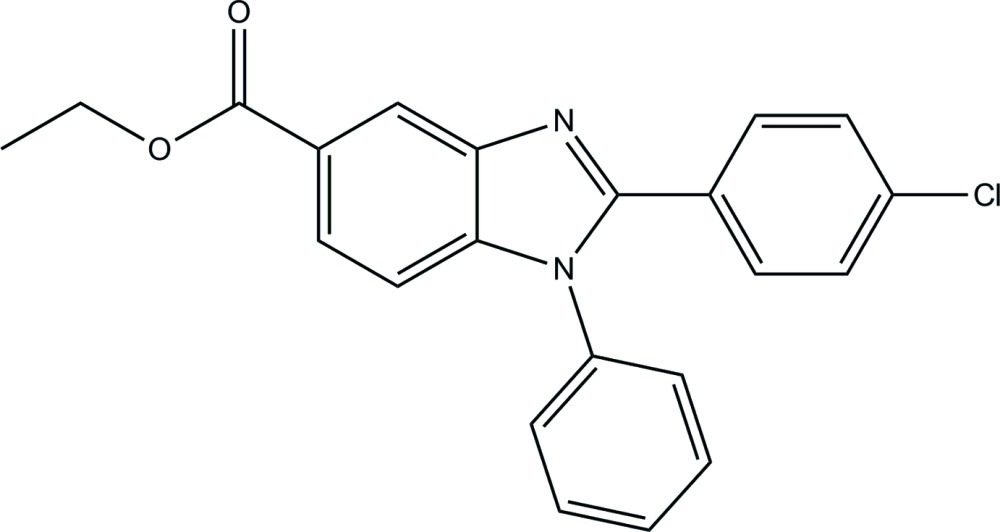


## Experimental

### 

#### Crystal data

C_22_H_17_ClN_2_O_2_
*M* *_r_* = 376.83
Triclinic, 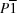

*a* = 9.3357 (2) Å
*b* = 9.7982 (2) Å
*c* = 11.7718 (4) Å
α = 107.502 (2)°
β = 102.106 (2)°
γ = 109.539 (1)°
*V* = 908.31 (4) Å^3^
*Z* = 2
Mo *K*α radiation
μ = 0.23 mm^−1^
*T* = 100 K
0.38 × 0.29 × 0.24 mm

#### Data collection

Bruker SMART APEXII CCD area-detector diffractometer
Absorption correction: multi-scan (*SADABS*; Bruker, 2009 ▶) *T*_min_ = 0.917, *T*_max_ = 0.946
18472 measured reflections
5326 independent reflections
4233 reflections with *I* > 2σ(*I*)
*R*_int_ = 0.033

#### Refinement

*R*[*F* ^2^ > 2σ(*F* ^2^)] = 0.048
*wR*(*F* ^2^) = 0.127
*S* = 1.04
5326 reflections
245 parameters
H-atom parameters constrained
Δρ_max_ = 0.57 e Å^−3^
Δρ_min_ = −0.31 e Å^−3^

### 

Data collection: *APEX2* (Bruker, 2009 ▶); cell refinement: *SAINT* (Bruker, 2009 ▶); data reduction: *SAINT*; program(s) used to solve structure: *SHELXTL* (Sheldrick, 2008 ▶); program(s) used to refine structure: *SHELXTL*; molecular graphics: *SHELXTL*; software used to prepare material for publication: *SHELXTL* and *PLATON* (Spek, 2009 ▶).

## Supplementary Material

Crystal structure: contains datablock(s) global, I. DOI: 10.1107/S1600536812022192/is5137sup1.cif

Structure factors: contains datablock(s) I. DOI: 10.1107/S1600536812022192/is5137Isup2.hkl

Supplementary material file. DOI: 10.1107/S1600536812022192/is5137Isup3.cml

Additional supplementary materials:  crystallographic information; 3D view; checkCIF report

## Figures and Tables

**Table 1:** Hydrogen-bond geometry (Å, °) *Cg*1, *Cg*2 and *Cg*3 are the centroids of the N1/C7/N2/C1/C6, C1–C6 and C8–C13 rings, respectively.

*D*—H⋯*A*	*D*—H	H⋯*A*	*D*⋯*A*	*D*—H⋯*A*
C15—H15*A*⋯O2^i^	0.95	2.39	3.324 (2)	166
C21—H21*A*⋯*Cg*1^ii^	0.99	2.63	3.5183 (16)	149
C12—H12*A*⋯*Cg*2^iii^	0.95	2.96	3.5940 (16)	125
C19—H19*A*⋯*Cg*3^iii^	0.95	2.60	3.4770 (18)	153

Symmetry codes: (i) 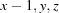; (ii) 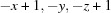; (iii) 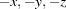.

**Fig. 1.**
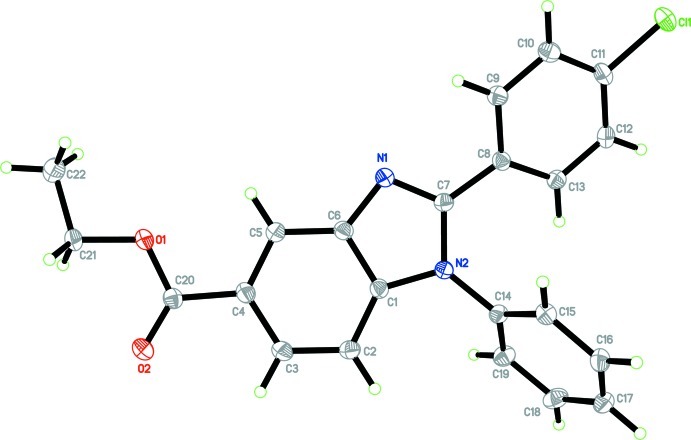
The molecular structure of the title compound, showing 50% probability displacement ellipsoids and the atom-numbering scheme.

**Fig. 2.**
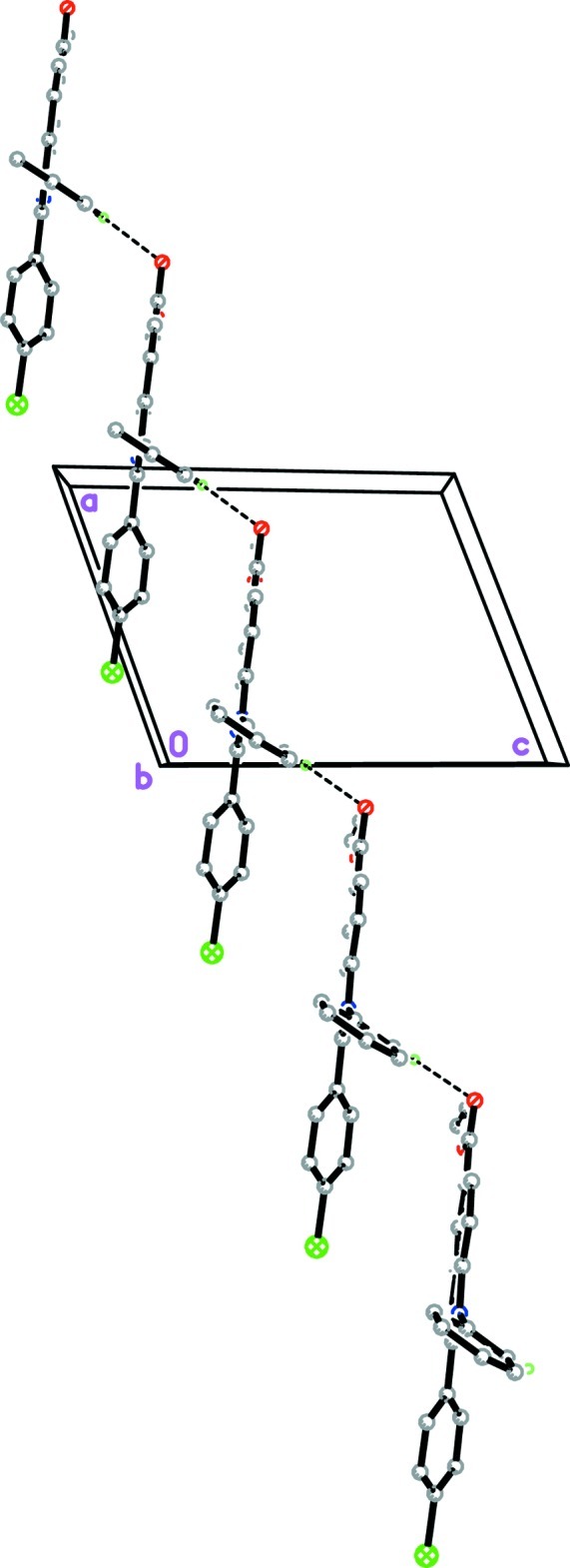
A crystal packing diagram of the title compound viewed along the b axis. The H atoms not involved in the intermolecular interactions (dashed lines) have been omitted for clarity.

## supplementary crystallographic information

### Comment

Substituted benzimidazoles are proven important drug leads. Substituted benzimidazole is the key building block for numerous compounds which plays crucial roles in the function of biologically important molecules (Tanious *et al.*, 2004). In particular, 2-substituted benzimidazoles are recognized as potential anticancer agents (Townsend & Revankar, 1970). As part of our ongoing structural studies of benzimidazole derivatives (Yoon *et al.*, 2011), we now report the structure of the title compound.

In the molecular structure (Fig. 1), The benzimidazole ring system (N1/N2/C1—C7) is essentially planar with a maximum deviation of 0.012 (2) Å at atom C1 and forms dihedral angles of 28.69 (6) and 63.65 (7)°, respectively, with the phenyl (C8–C13) and chloro-substituted benzene (C14–C19) ring. The dihedral angle between the phenyl ring and the chloro-substituted benzene ring is 64.23 (8)°. Bond lengths and angles are within normal ranges and are comparable to related structures (Yoon *et al.*, 2011; Kassim *et al.*, 2012; Yoon *et al.*, 2012).

The crystal packing is shown in Fig. 2. The molecules are linked into a zigzag chain along the *a*-axis *via* intermolecular C15—H15A···O2 (Table 1) hydrogen bonds. The crystal structure is further stabilized by intermolecular C21—H21A···*Cg*1, C1—H12A···*Cg*2 and C19—H19A···*Cg*3 (Table 1) interactions (*Cg*1, *Cg*2 and *Cg*3 are the centroids of N1/N2/C1/C6/C7, C1–C6 and C8–C13 rings, respectively).

### Experimental

Ethyl 3-amino-4-(phenyl amino) benzoate (0.84 mmol) and sodium metabisulfite adduct of chlorobenzaldehyde (1.68 mmol) were dissolved in DMF. The reaction mixture was reflux at 130 °C for 2 h. After completion, the reaction mixture was diluted in ethyl acetate (20 ml) and washed with water (20 ml). The organic layer was collected, dried over Na_2_SO_4_ and the evaporated in-vacuo to yield the product. The product was recrystallized from ethyl acetate.

### Refinement

All H atoms were positioned geometrically (C—H = 0.95–0.99 Å) and refined using a riding model with *U*_iso_(H) = 1.2 and 1.5*U*_eq_(C). A rotating group model was applied to the methyl group.

### Crystal data

**Table:**

C_22_H_17_ClN_2_O_2_	*Z* = 2
*M**_r_* = 376.83	*F*(000) = 392
Triclinic, *P*1	*D*_x_ = 1.378 Mg m^−^^3^
Hall symbol: -P 1	Mo *K*α radiation, λ = 0.71073 Å
*a* = 9.3357 (2) Å	Cell parameters from 7225 reflections
*b* = 9.7982 (2) Å	θ = 2.6–30.2°
*c* = 11.7718 (4) Å	µ = 0.23 mm^−^^1^
α = 107.502 (2)°	*T* = 100 K
β = 102.106 (2)°	Block, colourless
γ = 109.539 (1)°	0.38 × 0.29 × 0.24 mm
*V* = 908.31 (4) Å^3^	

### Data collection

**Table:**

Bruker SMART APEXII CCD area-detector diffractometer	5326 independent reflections
Radiation source: fine-focus sealed tube	4233 reflections with *I* > 2σ(*I*)
Graphite monochromator	*R*_int_ = 0.033
φ and ω scans	θ_max_ = 30.2°, θ_min_ = 1.9°
Absorption correction: multi-scan (*SADABS*; Bruker, 2009)	*h* = −13→13
*T*_min_ = 0.917, *T*_max_ = 0.946	*k* = −13→13
18472 measured reflections	*l* = −14→16

### Refinement

**Table:**

Refinement on *F*^2^	Primary atom site location: structure-invariant direct methods
Least-squares matrix: full	Secondary atom site location: difference Fourier map
*R*[*F*^2^ > 2σ(*F*^2^)] = 0.048	Hydrogen site location: inferred from neighbouring sites
*wR*(*F*^2^) = 0.127	H-atom parameters constrained
*S* = 1.04	*w* = 1/[σ^2^(*F*_o_^2^) + (0.0648*P*)^2^ + 0.3753*P*] where *P* = (*F*_o_^2^ + 2*F*_c_^2^)/3
5326 reflections	(Δ/σ)_max_ < 0.001
245 parameters	Δρ_max_ = 0.57 e Å^−^^3^
0 restraints	Δρ_min_ = −0.31 e Å^−^^3^

### Special details

**Table:**

Experimental. The crystal was placed in the cold stream of an Oxford Cryosystems Cobra open-flow nitrogen cryostat (Cosier & Glazer, 1986) operating at 100.0 (1) K.
Geometry. All e.s.d.'s (except the e.s.d. in the dihedral angle between two l.s. planes) are estimated using the full covariance matrix. The cell e.s.d.'s are taken into account individually in the estimation of e.s.d.'s in distances, angles and torsion angles; correlations between e.s.d.'s in cell parameters are only used when they are defined by crystal symmetry. An approximate (isotropic) treatment of cell e.s.d.'s is used for estimating e.s.d.'s involving l.s. planes.
Refinement. Refinement of *F*^2^ against ALL reflections. The weighted *R*-factor *wR* and goodness of fit *S* are based on *F*^2^, conventional *R*-factors *R* are based on *F*, with *F* set to zero for negative *F*^2^. The threshold expression of *F*^2^ > σ(*F*^2^) is used only for calculating *R*-factors(gt) *etc*. and is not relevant to the choice of reflections for refinement. *R*-factors based on *F*^2^ are statistically about twice as large as those based on *F*, and *R*- factors based on ALL data will be even larger.

### Fractional atomic coordinates and isotropic or equivalent isotropic displacement parameters (Å^2^)

**Table:**

	*x*	*y*	*z*	*U*_iso_*/*U*_eq_	
Cl1	−0.66895 (5)	−0.31206 (5)	−0.07003 (4)	0.02705 (11)	
O1	0.66316 (12)	−0.10624 (12)	0.40052 (10)	0.0196 (2)	
O2	0.84606 (13)	0.14573 (13)	0.46871 (11)	0.0229 (2)	
N1	0.11793 (15)	−0.10887 (14)	0.20878 (12)	0.0173 (2)	
N2	0.16095 (14)	0.14100 (14)	0.23348 (12)	0.0158 (2)	
C1	0.31182 (17)	0.14553 (17)	0.28366 (14)	0.0165 (3)	
C2	0.46660 (18)	0.26942 (17)	0.34187 (14)	0.0190 (3)	
H2A	0.4848	0.3744	0.3520	0.023*	
C3	0.59248 (18)	0.23218 (18)	0.38427 (15)	0.0201 (3)	
H3A	0.6997	0.3136	0.4249	0.024*	
C4	0.56507 (17)	0.07571 (17)	0.36849 (14)	0.0179 (3)	
C5	0.41041 (18)	−0.04706 (17)	0.31022 (14)	0.0180 (3)	
H5A	0.3924	−0.1521	0.2997	0.022*	
C6	0.28203 (17)	−0.01073 (17)	0.26755 (13)	0.0165 (3)	
C7	0.04967 (17)	−0.01571 (16)	0.19011 (13)	0.0159 (3)	
C8	−0.12637 (17)	−0.07740 (17)	0.12872 (13)	0.0158 (3)	
C9	−0.22379 (18)	−0.21369 (17)	0.13982 (14)	0.0178 (3)	
H9A	−0.1746	−0.2585	0.1881	0.021*	
C10	−0.39041 (19)	−0.28384 (18)	0.08158 (14)	0.0199 (3)	
H10A	−0.4559	−0.3739	0.0919	0.024*	
C11	−0.46027 (18)	−0.21990 (18)	0.00743 (14)	0.0192 (3)	
C12	−0.36672 (18)	−0.08703 (18)	−0.00704 (14)	0.0186 (3)	
H12A	−0.4161	−0.0458	−0.0588	0.022*	
C13	−0.19981 (18)	−0.01465 (17)	0.05489 (13)	0.0171 (3)	
H13A	−0.1354	0.0779	0.0470	0.021*	
C14	0.13273 (17)	0.27784 (17)	0.23909 (13)	0.0163 (3)	
C15	0.04178 (18)	0.32176 (18)	0.31023 (14)	0.0188 (3)	
H15A	−0.0040	0.2607	0.3534	0.023*	
C16	0.01898 (19)	0.45680 (19)	0.31695 (15)	0.0223 (3)	
H16A	−0.0417	0.4888	0.3661	0.027*	
C17	0.0840 (2)	0.54544 (18)	0.25259 (16)	0.0242 (3)	
H17A	0.0675	0.6374	0.2577	0.029*	
C18	0.1728 (2)	0.49899 (19)	0.18094 (16)	0.0246 (3)	
H18A	0.2161	0.5586	0.1360	0.029*	
C19	0.19904 (19)	0.36522 (18)	0.17450 (14)	0.0204 (3)	
H19A	0.2614	0.3343	0.1266	0.024*	
C20	0.70739 (18)	0.04619 (17)	0.41852 (14)	0.0171 (3)	
C21	0.79321 (18)	−0.14740 (18)	0.44432 (14)	0.0188 (3)	
H21A	0.8447	−0.0928	0.5378	0.023*	
H21B	0.8767	−0.1165	0.4056	0.023*	
C22	0.7175 (2)	−0.32370 (19)	0.40427 (16)	0.0254 (3)	
H22A	0.8013	−0.3578	0.4317	0.038*	
H22B	0.6667	−0.3759	0.3117	0.038*	
H22C	0.6354	−0.3523	0.4433	0.038*	

### Atomic displacement parameters (Å^2^)

**Table:**

	*U*^11^	*U*^22^	*U*^33^	*U*^12^	*U*^13^	*U*^23^
Cl1	0.01444 (18)	0.0278 (2)	0.0327 (2)	0.00672 (15)	0.00290 (15)	0.01040 (16)
O1	0.0137 (5)	0.0191 (5)	0.0244 (5)	0.0072 (4)	0.0042 (4)	0.0082 (4)
O2	0.0151 (5)	0.0228 (5)	0.0280 (6)	0.0061 (5)	0.0055 (4)	0.0100 (5)
N1	0.0149 (6)	0.0164 (6)	0.0196 (6)	0.0061 (5)	0.0041 (5)	0.0077 (5)
N2	0.0141 (6)	0.0151 (5)	0.0192 (6)	0.0060 (5)	0.0063 (5)	0.0079 (5)
C1	0.0151 (6)	0.0175 (6)	0.0181 (6)	0.0073 (6)	0.0064 (5)	0.0081 (5)
C2	0.0180 (7)	0.0154 (6)	0.0228 (7)	0.0060 (6)	0.0073 (6)	0.0078 (6)
C3	0.0152 (7)	0.0189 (7)	0.0237 (7)	0.0049 (6)	0.0067 (6)	0.0082 (6)
C4	0.0152 (7)	0.0201 (7)	0.0187 (7)	0.0073 (6)	0.0056 (5)	0.0084 (6)
C5	0.0169 (7)	0.0170 (6)	0.0206 (7)	0.0074 (6)	0.0064 (6)	0.0080 (5)
C6	0.0155 (6)	0.0163 (6)	0.0173 (6)	0.0062 (6)	0.0059 (5)	0.0067 (5)
C7	0.0163 (6)	0.0150 (6)	0.0156 (6)	0.0057 (6)	0.0053 (5)	0.0064 (5)
C8	0.0144 (6)	0.0159 (6)	0.0152 (6)	0.0062 (5)	0.0042 (5)	0.0045 (5)
C9	0.0177 (7)	0.0179 (6)	0.0172 (6)	0.0069 (6)	0.0057 (5)	0.0073 (5)
C10	0.0190 (7)	0.0202 (7)	0.0194 (7)	0.0066 (6)	0.0076 (6)	0.0080 (6)
C11	0.0140 (6)	0.0224 (7)	0.0180 (7)	0.0076 (6)	0.0047 (5)	0.0048 (6)
C12	0.0182 (7)	0.0217 (7)	0.0177 (7)	0.0114 (6)	0.0058 (5)	0.0071 (5)
C13	0.0183 (7)	0.0170 (6)	0.0176 (7)	0.0082 (6)	0.0072 (5)	0.0075 (5)
C14	0.0161 (6)	0.0151 (6)	0.0169 (6)	0.0065 (6)	0.0040 (5)	0.0066 (5)
C15	0.0172 (7)	0.0196 (7)	0.0190 (7)	0.0074 (6)	0.0058 (6)	0.0076 (6)
C16	0.0189 (7)	0.0221 (7)	0.0254 (8)	0.0111 (6)	0.0068 (6)	0.0067 (6)
C17	0.0225 (8)	0.0173 (7)	0.0295 (8)	0.0094 (6)	0.0027 (6)	0.0086 (6)
C18	0.0305 (8)	0.0183 (7)	0.0253 (8)	0.0089 (7)	0.0084 (7)	0.0119 (6)
C19	0.0240 (8)	0.0191 (7)	0.0202 (7)	0.0092 (6)	0.0099 (6)	0.0090 (6)
C20	0.0154 (6)	0.0198 (7)	0.0181 (7)	0.0078 (6)	0.0081 (5)	0.0083 (5)
C21	0.0154 (7)	0.0219 (7)	0.0198 (7)	0.0097 (6)	0.0049 (5)	0.0081 (6)
C22	0.0282 (8)	0.0247 (8)	0.0256 (8)	0.0139 (7)	0.0083 (7)	0.0110 (6)

### Geometric parameters (Å, º)

**Table:**

Cl1—C11	1.7393 (15)	C10—C11	1.394 (2)
O1—C20	1.3440 (17)	C10—H10A	0.9500
O1—C21	1.4466 (17)	C11—C12	1.386 (2)
O2—C20	1.2101 (18)	C12—C13	1.391 (2)
N1—C7	1.3186 (18)	C12—H12A	0.9500
N1—C6	1.3858 (19)	C13—H13A	0.9500
N2—C1	1.3885 (17)	C14—C19	1.389 (2)
N2—C7	1.3916 (18)	C14—C15	1.390 (2)
N2—C14	1.4359 (17)	C15—C16	1.391 (2)
C1—C2	1.394 (2)	C15—H15A	0.9500
C1—C6	1.4057 (19)	C16—C17	1.390 (2)
C2—C3	1.383 (2)	C16—H16A	0.9500
C2—H2A	0.9500	C17—C18	1.386 (2)
C3—C4	1.413 (2)	C17—H17A	0.9500
C3—H3A	0.9500	C18—C19	1.395 (2)
C4—C5	1.389 (2)	C18—H18A	0.9500
C4—C20	1.4923 (19)	C19—H19A	0.9500
C5—C6	1.3985 (19)	C21—C22	1.503 (2)
C5—H5A	0.9500	C21—H21A	0.9900
C7—C8	1.471 (2)	C21—H21B	0.9900
C8—C13	1.3990 (19)	C22—H22A	0.9800
C8—C9	1.403 (2)	C22—H22B	0.9800
C9—C10	1.384 (2)	C22—H22C	0.9800
C9—H9A	0.9500		
			
C20—O1—C21	115.83 (11)	C11—C12—H12A	120.3
C7—N1—C6	105.12 (12)	C13—C12—H12A	120.3
C1—N2—C7	106.08 (11)	C12—C13—C8	120.38 (14)
C1—N2—C14	124.39 (12)	C12—C13—H13A	119.8
C7—N2—C14	129.30 (12)	C8—C13—H13A	119.8
N2—C1—C2	131.98 (13)	C19—C14—C15	121.23 (13)
N2—C1—C6	105.41 (12)	C19—C14—N2	119.15 (13)
C2—C1—C6	122.60 (13)	C15—C14—N2	119.61 (13)
C3—C2—C1	116.80 (13)	C14—C15—C16	118.77 (14)
C3—C2—H2A	121.6	C14—C15—H15A	120.6
C1—C2—H2A	121.6	C16—C15—H15A	120.6
C2—C3—C4	121.44 (14)	C17—C16—C15	120.81 (14)
C2—C3—H3A	119.3	C17—C16—H16A	119.6
C4—C3—H3A	119.3	C15—C16—H16A	119.6
C5—C4—C3	121.36 (13)	C18—C17—C16	119.67 (14)
C5—C4—C20	120.65 (13)	C18—C17—H17A	120.2
C3—C4—C20	117.98 (13)	C16—C17—H17A	120.2
C4—C5—C6	117.73 (13)	C17—C18—C19	120.38 (15)
C4—C5—H5A	121.1	C17—C18—H18A	119.8
C6—C5—H5A	121.1	C19—C18—H18A	119.8
N1—C6—C5	129.46 (13)	C14—C19—C18	119.13 (14)
N1—C6—C1	110.46 (12)	C14—C19—H19A	120.4
C5—C6—C1	120.07 (13)	C18—C19—H19A	120.4
N1—C7—N2	112.93 (12)	O2—C20—O1	123.55 (13)
N1—C7—C8	121.50 (13)	O2—C20—C4	124.85 (13)
N2—C7—C8	125.57 (12)	O1—C20—C4	111.60 (12)
C13—C8—C9	118.95 (13)	O1—C21—C22	106.27 (12)
C13—C8—C7	124.14 (13)	O1—C21—H21A	110.5
C9—C8—C7	116.79 (12)	C22—C21—H21A	110.5
C10—C9—C8	121.07 (13)	O1—C21—H21B	110.5
C10—C9—H9A	119.5	C22—C21—H21B	110.5
C8—C9—H9A	119.5	H21A—C21—H21B	108.7
C9—C10—C11	118.76 (14)	C21—C22—H22A	109.5
C9—C10—H10A	120.6	C21—C22—H22B	109.5
C11—C10—H10A	120.6	H22A—C22—H22B	109.5
C12—C11—C10	121.37 (14)	C21—C22—H22C	109.5
C12—C11—Cl1	119.89 (11)	H22A—C22—H22C	109.5
C10—C11—Cl1	118.73 (12)	H22B—C22—H22C	109.5
C11—C12—C13	119.42 (13)		
			
C7—N2—C1—C2	−178.88 (15)	C13—C8—C9—C10	−1.5 (2)
C14—N2—C1—C2	−4.0 (2)	C7—C8—C9—C10	−177.72 (13)
C7—N2—C1—C6	−0.11 (15)	C8—C9—C10—C11	2.2 (2)
C14—N2—C1—C6	174.80 (13)	C9—C10—C11—C12	−1.1 (2)
N2—C1—C2—C3	178.50 (15)	C9—C10—C11—Cl1	177.86 (11)
C6—C1—C2—C3	−0.1 (2)	C10—C11—C12—C13	−0.8 (2)
C1—C2—C3—C4	0.3 (2)	Cl1—C11—C12—C13	−179.71 (11)
C2—C3—C4—C5	−0.3 (2)	C11—C12—C13—C8	1.5 (2)
C2—C3—C4—C20	−179.22 (14)	C9—C8—C13—C12	−0.4 (2)
C3—C4—C5—C6	−0.1 (2)	C7—C8—C13—C12	175.52 (13)
C20—C4—C5—C6	178.85 (13)	C1—N2—C14—C19	65.11 (19)
C7—N1—C6—C5	179.06 (15)	C7—N2—C14—C19	−121.21 (16)
C7—N1—C6—C1	−0.26 (16)	C1—N2—C14—C15	−114.00 (16)
C4—C5—C6—N1	−178.95 (14)	C7—N2—C14—C15	59.7 (2)
C4—C5—C6—C1	0.3 (2)	C19—C14—C15—C16	−0.6 (2)
N2—C1—C6—N1	0.23 (16)	N2—C14—C15—C16	178.49 (13)
C2—C1—C6—N1	179.15 (13)	C14—C15—C16—C17	0.8 (2)
N2—C1—C6—C5	−179.16 (13)	C15—C16—C17—C18	−0.1 (2)
C2—C1—C6—C5	−0.2 (2)	C16—C17—C18—C19	−0.8 (2)
C6—N1—C7—N2	0.19 (16)	C15—C14—C19—C18	−0.3 (2)
C6—N1—C7—C8	179.89 (13)	N2—C14—C19—C18	−179.41 (14)
C1—N2—C7—N1	−0.05 (16)	C17—C18—C19—C14	1.0 (2)
C14—N2—C7—N1	−174.62 (13)	C21—O1—C20—O2	−0.5 (2)
C1—N2—C7—C8	−179.73 (13)	C21—O1—C20—C4	179.16 (12)
C14—N2—C7—C8	5.7 (2)	C5—C4—C20—O2	178.84 (15)
N1—C7—C8—C13	−149.37 (15)	C3—C4—C20—O2	−2.2 (2)
N2—C7—C8—C13	30.3 (2)	C5—C4—C20—O1	−0.83 (19)
N1—C7—C8—C9	26.6 (2)	C3—C4—C20—O1	178.10 (13)
N2—C7—C8—C9	−153.74 (14)	C20—O1—C21—C22	−175.16 (12)

### Hydrogen-bond geometry (Å, º)

*Cg*1, *Cg*2 and *Cg*3 are the centroids of the N1/C7/N2/C1/C6, C1–C6 and C8–C13 rings, respectively.

**Table:**

*D*—H···*A*	*D*—H	H···*A*	*D*···*A*	*D*—H···*A*
C15—H15*A*···O2^i^	0.95	2.39	3.324 (2)	166
C21—H21*A*···*Cg*1^ii^	0.99	2.63	3.5183 (16)	149
C12—H12*A*···*Cg*2^iii^	0.95	2.96	3.5940 (16)	125
C19—H19*A*···*Cg*3^iii^	0.95	2.60	3.4770 (18)	153

Symmetry codes: (i) *x*−1, *y*, *z*; (ii) −*x*+1, −*y*, −*z*+1; (iii) −*x*, −*y*, −*z*.
